# A Fluorescence-Based Genetic Screen to Study Retinal Degeneration in *Drosophila*

**DOI:** 10.1371/journal.pone.0144925

**Published:** 2015-12-14

**Authors:** Yu Huang, Jun Xie, Tao Wang

**Affiliations:** 1 National Institute of Biological Sciences, Beijing, China; 2 College of Biological Sciences, China Agricultural University, Beijing, China; Ecole Normale Supérieure, FRANCE

## Abstract

The *Drosophila* visual system has been proved to be a powerful genetic model to study eye disease such as retinal degeneration. Here, we describe a genetic method termed “*Rh1*::*GFP ey-flp/hid*” that is based on the fluorescence of GFP-tagged major rhodopsin Rh1 in the eyes of living flies and can be used to monitor the integrity of photoreceptor cells. Through combination of this method and ERG recording, we examined a collection of 667 mutants and identified 18 genes that are required for photoreceptor cell maintenance, photoresponse, and rhodopsin synthesis. Our findings demonstrate that this “*Rh1*::*GFP ey-flp/hid*” method enables high-throughput F1 genetic screens to rapidly and precisely identify mutations of retinal degeneration.

## Introduction

The *Drosophila* visual system has been shown to be proven to be a powerful genetic model for dissecting the molecular mechanisms underlying retinal degeneration and G-protein-coupled signaling cascades. Mutations in most genes that functions in phototransduction result in light-dependent photoreceptor cell death. Therefore, genetic screens in *Drosophila* could isolate mutations of many genes involved in retinal degeneration and could deepen our understanding of their counterpart genes in human diseases.

*Drosophila* phototransduction offers the opportunity to combine classical and modern genetic approaches to identify genes and proteins that function in phototransduction and/or that are required for photoreceptor cell survival [1–4]. Electroretinogram recordings (ERGs) are among the analysis tools that have driven the progress of *Drosophila* phototransduction research; this technique is simple enough to be used to perform genetic screens [3]. However, due to the requirement of fixing animals, flies can not survive after ERG assay, which makes it less suitable for mutagenesis F1 screens.

In addition, most retinal degeneration mutations in *Drosophila* were originally identified from photoresponse-based screens, which do not fully represent the complexity of retinal degeneration diseases in human. Moreover, classic screens of adult animals for aberrant phototransduction and eye morphology often cannot isolate essential genes involved in these pathways since such genes are often indispensable for organism viability. A few mosaic methods have been developed that make an entire eye homozygous for a mutation [5, 6]. Large scale screens for neurotransmission and phototransduction mutants have been conducted based on these methods [7, 8]. However, phototaxis in the F1 generation is not sensitive enough, and the ERG-based high throughput screening is time-consuming [8–10].

Given these nontrivial limitations, we were motivated to develop a fluorescence-based “*Rh1*::*GFP ey-flp/hid*” method for F1 screening of retinal degeneration mutants. This method is based on a modified *EGUF/hid* technique to generate eyes of homozygous mutations and uses GFP-tagged Rh1 (major rhodopsin) as a marker for photoreceptor cell integrity. Using this *Rh1*::*GFP ey-flp/hid* method, we screened the UCLA URCFG P-element recessive lethal collection, and identified several types of mutations affecting photoreceptor cell survival, phototransduction, and rhodopsin homeostasis.

## Results

### Development of the *Rh1*::*GFP ey-flp/hid* screening method

**Fig 1:**
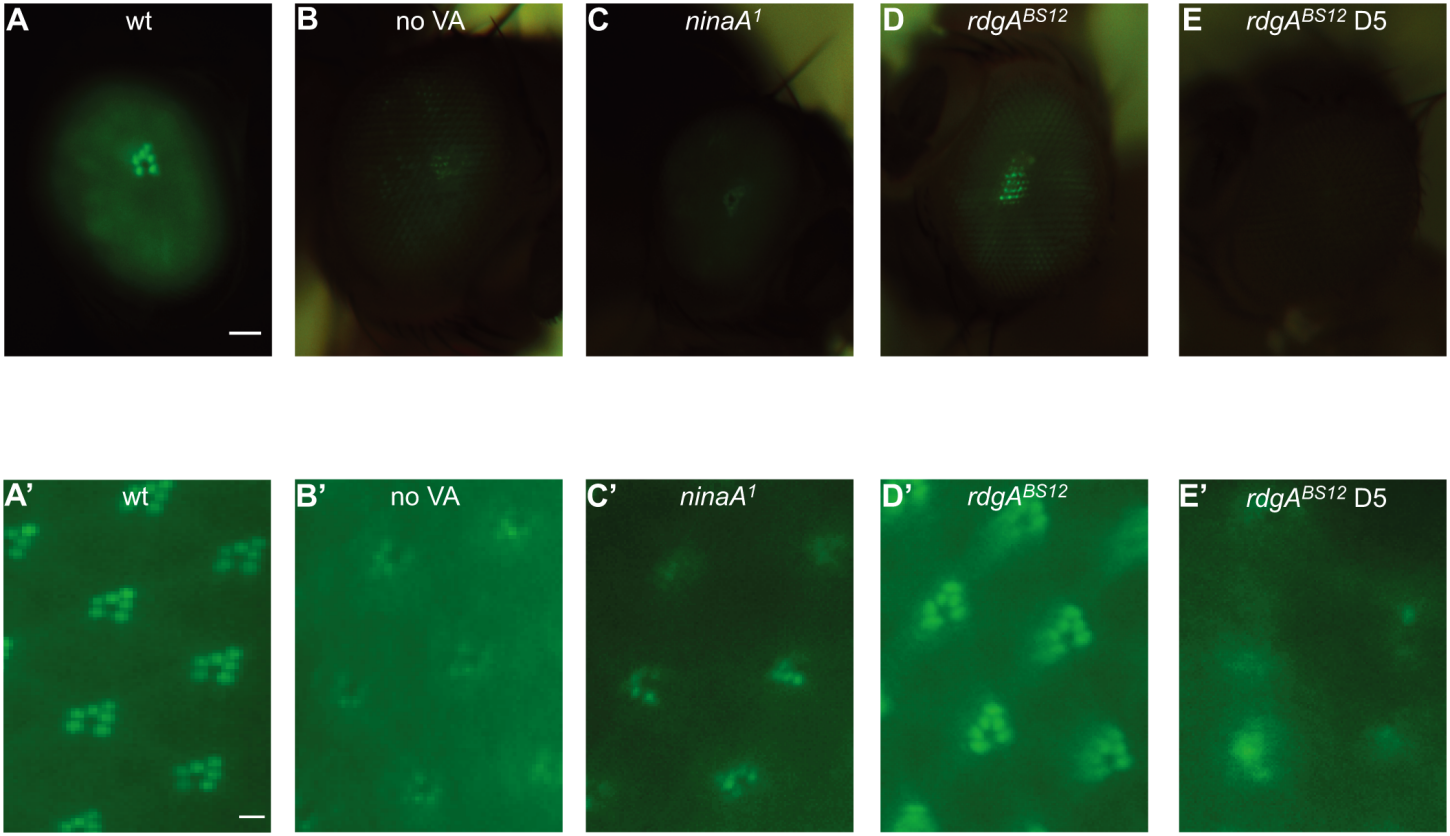
Rhodopsin levels and the integrity of photoreceptor cells using Rh1-GFP. Representative images of the GFP fluorescence in intact eyes are shown. (A-E) The green fluorescing deep pseudopupil of flies with different genotypes expressing Rh1-GFP (upper panel). (A’-E’) GFP-fluorescence was detected in intact eyes after cornea optical neutralization by water immersion. (A, A’) wild type (*Rh1*::*GFP*), (B, B’) *Rh1*::*GFP* flies raised in vitamin A-free food, (C, C’) *ninaA* ^*1*^ (*Rh1*::*GFP;ninaA* ^*1*^), (D, D’) *rdgA* ^*BS12*^ (*rdgA* ^*BS12*^ *Rh1*::*GFP*), (E, E’) *rdgA* ^*BS12*^ 5 day-old. With the exception of the *rdgA* ^*BS12*^ flies in E and E’, flies depicted in this figure were 1 day old. Scale bar on upper panels, 50 μm; on lower panel, 2 μm.

To monitor the integrity of photoreceptor cells in live animals, we generated *Rh1*::*GFP* transgenic flies, which express a GFP-tagged major rhodopsin Rh1 protein in R1-6 photoreceptor cells under the control of the *ninaE* (*rh1*) promotor. Under a fluorescence microscope, regular *Rh1*::*GFP* compound eyes showed an intensely green fluorescing deep pseudopupil. This fluorescence signal was markedly reduced in flies raised on vitamin A-free food as well as in *ninaA* ^*1*^ mutant flies with disrupted Rh1 biosynthesis. It was also reduced in the *rdgA* ^*BS12*^ mutant background which caused a rapid retinal degeneration (Fig 1A–1E). Since the fluorescing Rh1-GFP pseudopupil can be observed in living flies and because it represents the Rh1 levels and/or rhabdomere structures, it is ideally suited for a use in a high throughput genetic screen. Photoreceptor cell integrity, indicated by the number of Rh1 GFP-tagged rhabdomeres, was further visualized at a higher resolution following cornea optical neutralization using fluorescence microscopy with oil-immersion objectives [11]. Compared with wild type, which had intensive GFP fluorescence for 6 rhabdomeres, the GFP signals were dramatically reduced in rhabdomeres of flies raised on vitamin A-free food and in *ninaA* ^1^ mutant (Fig 1A’–1C’). Rhabdomere structures were lost in the retina of *rdgA* ^*BS12*^ flies at 5 days (Fig 1D’ and 1E’). Therefore, fluorescence of *Rh1*::*GFP* is a good marker for rhodopsin levels and is suitable for use in screens targeting mutants of retinal degeneration.

**Fig 2:**
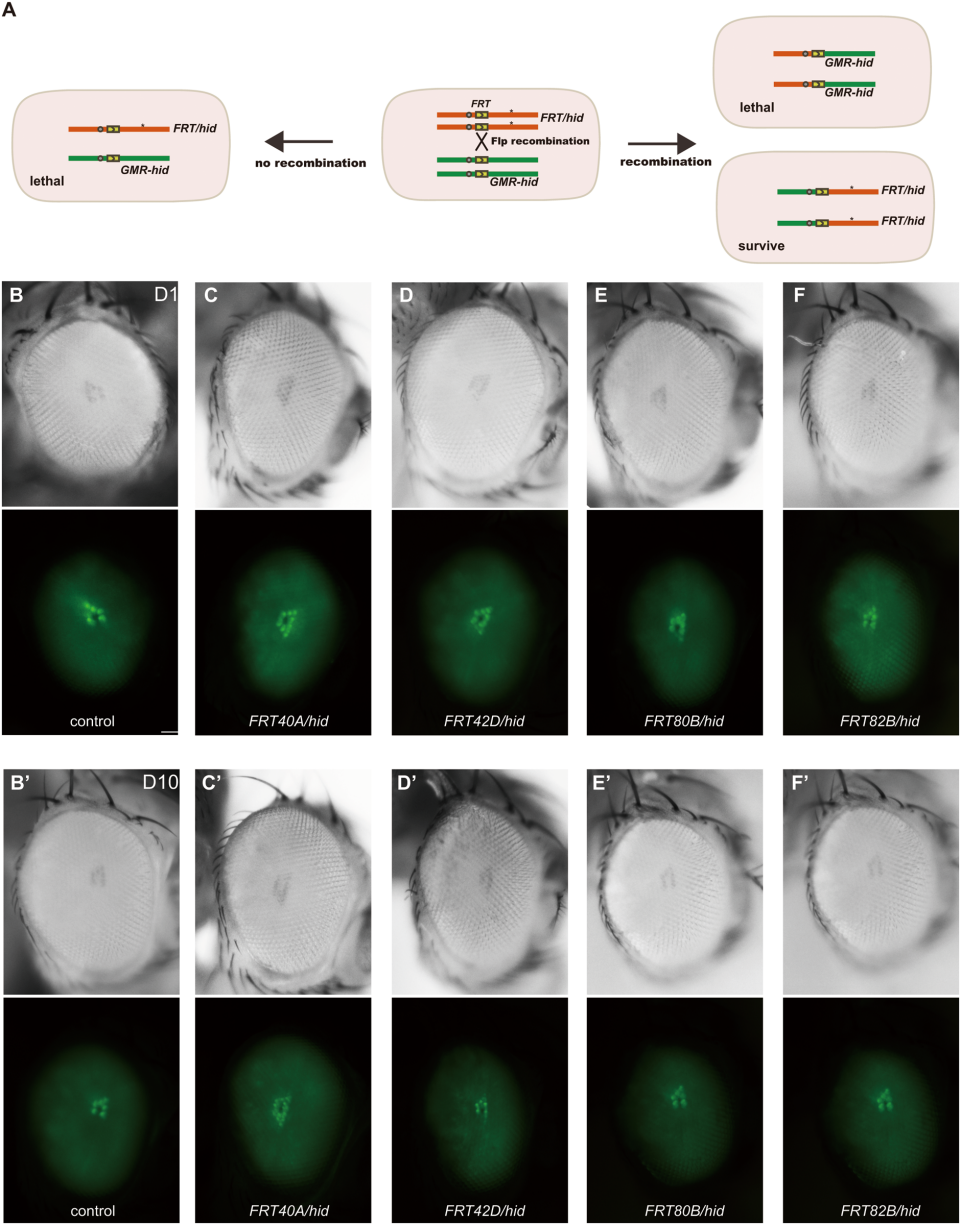
Application of the *ey-flp/hid* method in *Rh1*::*GFP* background fly. (A) Schematic diagram of mitotic recombination occurring in eye precursor cells with the *Rh1*::*GFP ey-flp/hid* method. Only retina cells that are homozygous for the FRT chromosome carrying a mutation (*) survive, because *GMR-hid* dominantly causes lethality of retina cells. (B-F) *Drosophila* eyes representative of the following genotypes are shown in light (upper panels) or fluorescence (lower panels) images: (B, B’) control: *ey-flp Rh1*::*GFP;FRT40A*, (C, C’) *FRT40A/hid* (*ey-flp Rh1*::*GFP;FRT40A/GMR-hid CL FRT40A*), (D, D’) *FRT42d/hid* (*ey-flp Rh1*::*GFP;FRT42D/FRT42D GMR-hid CL*), (E, E’) *FRT80B/hid* (*ey-flp Rh1*::*GFP;FRT80B/GMR-hid CL FRT80B*), (F, F’) *FRT82B/hid* (*ey-flp Rh1*::*GFP;FRT82B/ FRT82B GMR-hid CL*). 1 day-old flies were used in A-E. 10 day-old flies were used in A’-E’. Flies were raised under a 12 hr light/12 hr dark cycle. Scale bar, 50 μm.

The *EGUF/hid* technique can generate eyes homozygous for a mutant allele in an otherwise heterozygous background [5]. This system employed the *GAL4/UAS* and *FLP/FRT* systems to induce mitotic recombination of *FRT* bearing chromosome arms specifically in the eye by combination of *ey-GAL4*/*UAS-FLP*, and the dominant photoreceptor-specific cell-lethal transgene *GMR-hid* and a recessive cell lethal (*CL*) mutation to eliminate all photoreceptors during development in which the desired chromosome arm has not been made homozygous. Thus, the mutant phenotype is recognized in the F1 generation, and mutations in essential genes can be recovered because the mutation is homozygous solely in the eye, making this screen potentially quite powerful. In combination with the *Rh1*::*GFP* transgene, this adult recombinant eye technique can facilitate high-throughput screening of candidate mutations. We also modified the technique by using *ey-flp* to express FLP directly under the *ey* promoter instead of *ey-GAL4/UAS-FLP* to simplify the manipulation. As well as the *EGUF/hid* system, normally-developed fully homozygous mutant eyes in otherwise heterozygous animals were generated with the *ey-flp/hid* system in 4 *FRT* arms, including *FRT40A*, *FRT42D*, *FRT80B*, and *FRT82B* (Fig 2). Moreover, upon introducing *Rh1*::*GFP*, fluorescing deep pseudopupils in mosaic animals were similar to those of the wild-type at both day1 and day 10, indicating that *Rh1*::*GFP* does not affect photoreceptor morphology (Fig 2).

### Testing the *Rh1*::*GFP ey-flp/hid* method on mutants of rhodopsin homeostasis, retinal degeneration, and phototransduction

**Fig 3:**
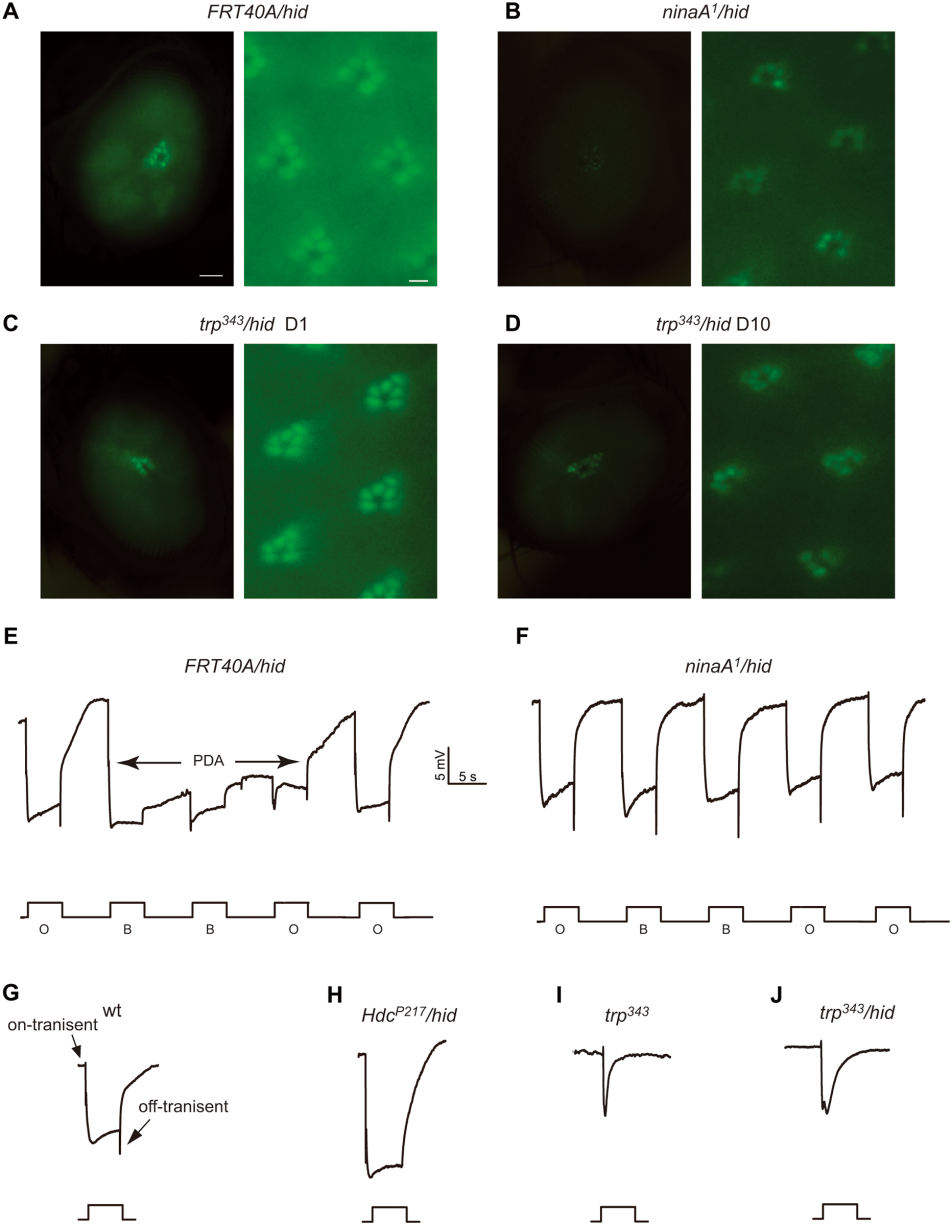
Analysis of mutants of rhodopsin homeostasis, retinal degeneration, and phototransduction with the *Rh1*::*GFP ey-flp/hid* method. (A-E) Detection of fluorescence in the deep pseudopupil (left panels) and by cornea optical neutralization (right panel). (A) *FRT40A/hid*, (B) *ninaA* ^*1*^ */hid* (*ey-flp Rh1*::*GFP;ninaA* ^*1*^ *FRT40A/GMR-hid CL FRT40A*), (C) *trp* ^*P343*^ */hid* (*ey-flp Rh1*::*GFP;FRT82B trp* ^*P343*^ */ FRT82B GMR-hid CL*), (D) *trp* ^*P343*^ */hid* 10 day-old. 1 day-old flies were used, with the exception of the *trp* ^*P343*^ */hid* flies, which were 10 day-old (D). Scale bar in right panels, 50 μm; in the left panels, 2μm. (E-H) ERG recordings of (E) wild type and (F) *ninaA* ^*1*^ */hid* flies. Flies were exposed to 5 s pulses of orange light (O) or blue light (B), interspersed by 7 s, as indicated. A PDA was induced in the wild-type by blue light and terminated by orange light (arrows). (G-J) ERG response of (G) wild-type, (H) *Hdc* ^*P217*^ */hid*, (I) *trp* ^*P343*^, and (J) *trp* ^*P343*^ */hid* flies in response to a 5-s orange light stimulus.

To determine whether the photoreceptors in the recombinant eyes generated via the *ey-flp/hid* system were capable of representing mutant phenotypes, we next generated recombinant eyes of a few mutant genes using the *Rh1*::*GFP ey-flp/hid* system and compared the recombinant mutant eyes with wild-type recombinant eyes. The GFP fluorescence was severely reduced in the *ninaA* ^*1*^ mutant recombinant eye (*ninaA* ^*1*^/*hid*), a result that correlates with the endogenous Rh1 levels [12, 13] (Fig 3A and 3B). We further checked the visual response by performing ERG recordings, which are extracellular recordings that measure the summed responses of all retinal cells to light. In response to orange light, wild-type flies display a rapid corneal negative response that quickly returns to baseline levels after cessation of the light stimulation. After exposure to blue light, there is a prolonged depolarization afterpotential (PDA) that is due to an excessive accumulation of Rh1 in an active form (Fig 3E). Thus, in *ninaA* ^*1*^ mutant flies, a PDA is not produced due to a large reduction of Rh1; and the same no PDA phenotype was recorded in the *ninaA* ^*1*^ */hid* animals (Fig 3F).

Given that Rh1-GFP also marks rhabdomeres and indicates the integrity of photoreceptor cells, we checked if the *Rh1*::*GFP ey-flp/hid* system could be used to monitor mutations that lead to retinal degeneration by generating recombinant eyes for a *trp* mutant (*trp* ^*343*^ */hid*). The *trp* gene encodes a major ion channel in phototransduction, and mutations of *trp* result in transient receptor potential light responses and retinal degeneration [14, 15]. Similarly as in homozygous *trp* ^*343*^ mutant animals, the ERG phenotype of the *trp* ^*343*^ */hid* flies was characterized by a transient response to light (Fig 3I and 3J). Moreover, the *trp* ^*343*^ */hid* flies underwent an age-dependent loss of Rh1-GFP signals (Fig 3C and 3D). At the age of 10 days, the GFP signals were dramatically reduced in the *trp* ^*343*^ */hid* eyes, and fewer fluorescing rhabdomeres were detected by cornea optical neutralization, which is consistent with light dependent retinal degeneration in *trp* ^*343*^ mutant animals (Fig 3D) [15].

Except the remained depolarization arising from responses of all retinal cells, the ERG diagram also includes on- and off-transients emanating from activity in the second-order neurons in the lamina (Fig 3F). The mutations with defective synaptic transmission or synapse development could result in preferential reduction in on- and off-transients. Mutations in the *Hdc* gene, which encodes a histidine decarboxylase for generating the photoreceptor cell neurotransmitter histamine, cause reduced histamine and loss of on- and off-transients in the ERG paradigm [16]. Although homozygous *Hdc* ^*P217*^ eyes (*Hdc* ^*P217*^ */hid*) showed no Rh1-GFP fluorescence change, the *Hdc* ^*P217*^ */hid* flies lost on- and off- transients during light response (Fig 3H). These data suggested that the *Rh1*::*GFP ey-flp/hid* system can be used for high throughput screening for mutations that cause defects in rhodopsin homeostasis and retinal degeneration.

### Genetic screen combining the *Rh1*::*GFP ey-flp/hid* method and ERG recording

To further test the *Rh1*::*GFP ey-flp/hid* system as an advanced genetic screen to isolate mutations regulating rhodopsin levels and photoreceptor cell integrity, we screened the P-element recessive lethal lines inserted in both arms of the second and third chromosomes from the UCLA URCFG collection [17]. This collection has been used for screening of genes required for photoreceptor cell development, cell survival, and rhodopsin localization [18–21]. Therefore, we only counted flies with normal morphology of compound eyes at 1 day old to avoid mutants affecting retina development and general cell survival. We analyzed insertion lines on the second or third chromosome carrying FRT sequences (667 independent lines) (S1 Table). By performing fluorescence deep pseudopupil assays and confirming with corneal optical neutralization at both day 1 and day 10, as well as ERG recording at day 1, we identified 18 lines with defects in Rh1 fluorescence at day 10 or defective ERG at day 1 (Table 1). Among these 18 lines, 14 lines showed loss of fluorescence deep pseudopupil at day 10, and these 14 retinal degeneration mutants included 4 lines with normal ERG and Rh1 levels, 1 line with Rh1 reduction, and 9 lines with ERG defect (Table 1). The remaining 4 lines only had ERG defects without Rh1-GFP fluorescence loss even at age of 20 days.

**Table 1:** Retinal degeneration and photoresponse defective mutants.

Gene name	Phenotype			Function putative
	Degeneration	ERG	Rh1	
*CG30415*	degeneration	wt	decrease	unknown
*Tps1*	degeneration	wt	wt	alpha, alpha-trehalose-phosphate synthase (UDP-forming) activity
*dup*	degeneration	wt	wt	DNA binding, glial cell development
*Lis-1*	degeneration	wt	wt	dynein binding; enzyme regulator activity
*dve*	degeneration	wt	wt	sequence-specific DNA binding transcription factor activity
*ifc*	degeneration	small response	wt	sphingolipid delta-4 desaturase activity
*sick*	degeneration	small response	wt	ATP binding
*srp54*	degeneration	small response	wt	mRNA binding
*chn*	degeneration	small response	wt	sequence-specific DNA binding transcription factor activity
*aats-val*	degeneration	no off-transient	wt	glutamate-tRNA ligase activity; valine-tRNA ligase activity
*dmn*	degeneration	no off-transient	wt	positive regulation of retrograde axon cargo transport
*rab6*	degeneration	no off-transient	wt	regulation of postsynaptic membrane potential
*Scox*	degeneration	slow termination	wt	cytochrome-c oxidase activity, copper chaperone activity
*porin*	degeneration	slow termination	wt	regulation of anion transport on mitochondria
*mmp2*	wt	small response	wt	metalloendopeptidase activity
*milt*	wt	no off-transient	wt	axon transport of mitochondrion
*synj*	wt	no off-transient	wt	inositol trisphosphate phosphatase activity
opa1-like	wt	no off-transient	wt	GTPase in mitofission

### Light-dependent retinal degeneration in the *scox* and *porin* mutant flies

**Fig 4:**
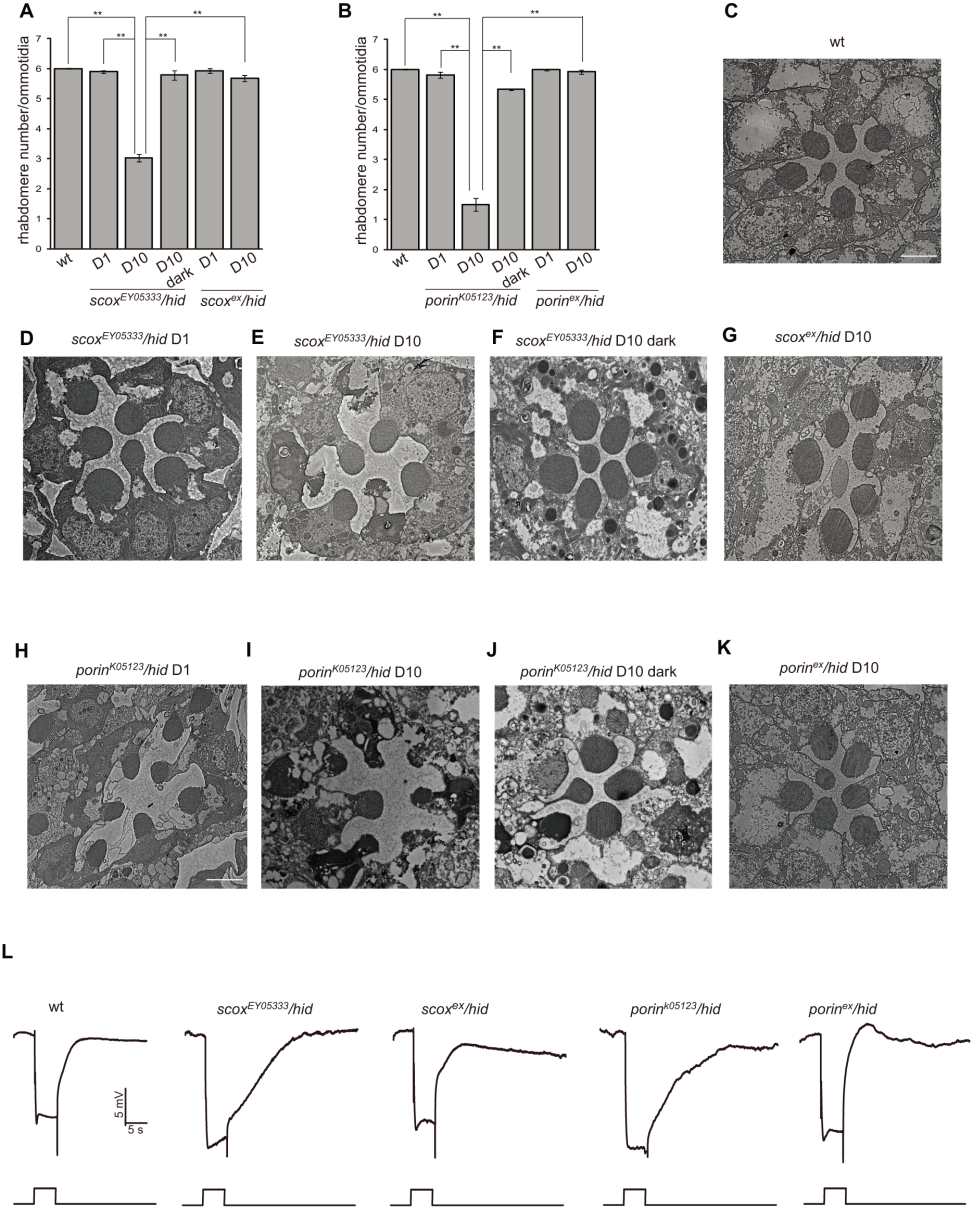
The *scox* and *porin* mutations lead to light-dependent photoreceptor cell degeneration. (A-B) Average rhabdomere numbers per ommatidia of (A) the *scox* mutant flies and (B) the *porin* mutant flies under the indicated conditions. Each data point was based on examination of >60 ommatidia from >3 flies. Error bars represent the SD. Asterisks indicate statistically-significant differences (one-way ANOVA and post-hoc Dunnett’s test, **p < 0.01). (C-K) Transmission electron microscopy sections of single ommatidia of fly compound eyes with the indicated genotype and conditions. (C) 10 day-old wild-type, (D) 1 day-old *scox* ^*EY05333*^ */hid* (*ey-flp Rh1*::*GFP; scox* ^*EY05333*^ *FRT40A/GMR-hid CL FRT40A*), (E) 10 day-old *scox* ^*EY05333*^ */hid*, (F) 10 day-old *scox* ^*EY05333*^ */hid* under dark condition, (G) 10 day-old P-element excised *scox* ^*ex*^ */hid* (*ey-flp Rh1*::*GFP; scox* ^*ex*^ *FRT40A/GMR-hid CL FRT40A*), (H) 1 day-old *porin* ^*k05123*^ */hid* (*ey-flp Rh1*::*GFP; porin* ^*k05123*^ *FRT40A/GMR-hid CL FRT40A*), (I) 10 day-old *porin* ^*k05123*^ */hid*, (J) 10 day-old *porin* ^*k05123*^ */hid* under dark condition, (K) 10 day-old p-element excised *porin* ^*ex*^ */hid* (*ey-flp Rh1*::*GFP; porin* ^*ex*^ *FRT40A/GMR-hid CL FRT40A*). Scale bar, 2 μm. With the exception of the dark-reared (F) *scox* ^*EY05333*^ */hid* and (J) *porin* ^*k05123*^ */hid* flies, flies were maintained under a 12 hr light/12 hr dark cycle. (L) ERG responses of wild-type, *scox* ^*EY05333*^ */hid*, *scox* ^*ex*^ */hid*, *porin* ^*k05123*^ */hid*, and p-element excised *porin* ^*ex*^ */hid* flies in response to a 10-s orange light stimulus as indicated. Flies used were less than 2 days old.

Among the 14 mutations with retinal degeneration phenotypes, two genes, including *scox* and *porin*, have putative functions in mitochondria. The *scox* gene encodes the *Drosophila* homologue of SCO (Synthesis of Cytochrome c Oxidase), which is a copper-donor chaperone required for the assembly of mitochondrial cytochrome c oxidase (COX) [22]. The *scox* ^*EY05333*^ recombinant eye had normal Rh1-GFP fluorescence right after eclosure, but underwent an age- and light-dependent loss of fluorescence and rhabdomeres (Fig 4A). This age-dependent retinal degeneration was caused by disruption of *scox* by P-element *EY05333*, as precise excision of *scox* ^*EY05333*^ (*scox* ^*ex*^) rescued this loss of fluorescence (Fig 4A). The results obtained with the fluorescence deep pseudopupil and cornea optical neutralization methods were confirmed by examining the morphology of *scox* ^*EY05333*^ retinas by transmission electron microscopy (Fig 4C–4G). The ommatidia from wild-type compound eyes contained the full set of seven intact rhabdomeres at all ages (Fig 4C). Few rhabdomeres were detected in 10-day-old *scox* ^*EY05333*^ ommatidia, although seven intact rhabdomeres were present at day 1 (Fig 4D and 4E). Moreover, both the dark-raised *scox* ^*EY05333*^ and *scox* ^*ex*^ flies raised under a normal light/dark cycle contained the full complement of seven rhabdomeres (Fig 4F and 4G). Since the human homologs of SCOX, Sco I, and Sco II are required for the assembly of mitochondrial respiratory complex IV, we tested the COX chaperon function of SCOX [23]. Using the *hs-FLP/FRT* system, we made homozygous *scox* mutant cell clones in heterozygous tissues that were marked by the absence of red fluorescent protein (RFP) [24]. By staining eye imaginal discs with anti-CoIV and anti-Tom20 antibodies, the cellular levels of COX and whole mitochondria were evaluated. As results, the levels of CoIV, but not Tom20, were reduced in the *scox* ^*EY05333*^ mutant cells, indicating that the loss of *scox* in cells specifically reduced COX levels without affecting the overall amount of mitochondria (S1 Fig). It is therefore clear that the *scox* mutations disrupt cytochrome c oxidase assembly, thereby reducing the COX complex levels in the mutant cells.

The *Drosophila porin* gene encodes a major Voltage-dependent anion channel (VDAC), which is an outer mitochondrial membrane component of mitochondrial permeability transition pores (PTP). Porin plays an important role in regulating energy metabolism and apoptosis by mediating the transport of ions and metabolites across the mitochondrial outer membrane [25]. *porin* ^*k05123*^ mutant eyes gradually lost Rh1-GFP and rhabdomere fluorescence after eclosure, and remobilization of the P-element for the *porin* ^*k05123*^ mutant (*porin* ^*ex*^) rescued the age-dependent retinal degeneration phenotype (Fig 4B). The results obtained with the fluorescence deep pseudopupil and cornea optical neutralization methods were confirmed by examining the morphology of *porin* ^*k05123*^ retinas with TEM (Fig 4H–4K). Both of the retinal degeneration phenotypes caused by the scox and *porin* mutations were light-dependent, as no significant fluorescence or morphological changes were detected in the dark-raised animals (Fig 4F and 4J). The scox and *porin* mutations may therefore affect phototransduction. It has been reported that the *porin* mutant animals displayed an inactivation ERG phenotype [26]. We next examined the ERG response of both *scox* and *porin* mutant eyes. Both *scox* ^*EY05333*^ and *porin* ^*k05123*^ mutant eyes displayed slower termination of the light response relative to wild-type, and *scox* ^*ex*^ and *porin* ^*ex*^ eyes reversed this slower termination phenotype, suggesting that mitochondria play an important role in light response termination (Fig 4L).

### Dmn and Rab6 are required for synaptic transmission

**Fig 5:**
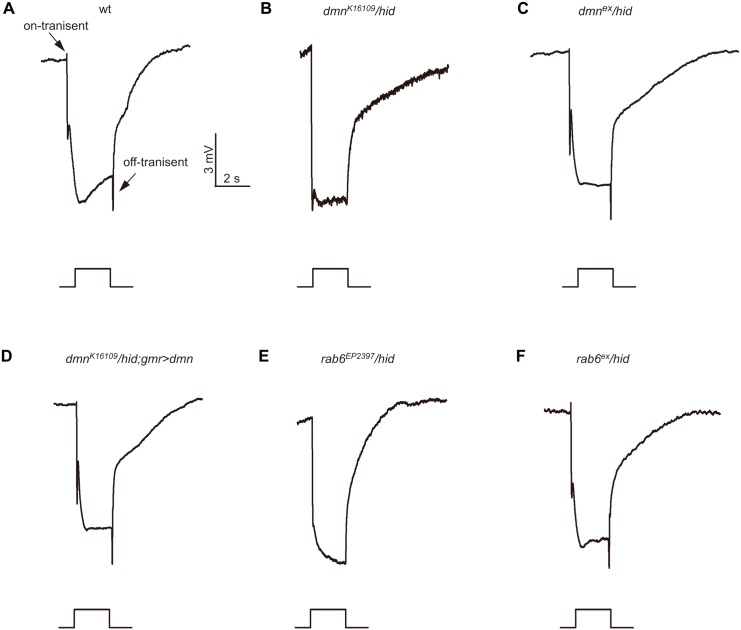
Reduced on- and off-transients in *dmn* and *rab6* mutants. ERG response of (A) control, (B) *dmn* ^*K16109*^ */hid* (*ey-flp Rh1*::*GFP; FRT42D dmn* ^*K16109*^ */ FRT42D GMR-hid CL*), (C) precise p-element excised *dmn* ^*ex*^ */hid* (*ey-flp Rh1*::*GFP; FRT42D dmn* ^*ex*^ */ FRT42D GMR-hid CL*), (D) *dmn* ^*K16109*^ */hid;GMR>dmn* (*ey-flp Rh1*::*GFP; FRT42D dmn* ^*K16109*^ */ FRT42D GMR-hid CL;GMR-gal4/UAS-dmn*), (E) *rab6* ^*EP2397*^ */hid* (*ey-flp Rh1*::*GFP; rab6* ^*EP2397*^ *FRT40A/GMR-hid CL FRT40A*), (F) precise p-element excised *rab6* ^*ex*^ */hid* (*ey-flp Rh1*::*GFP; rab6* ^*ex*^ *FRT40A/GMR-hid CL FRT40A*) flies in response to a 2-s orange light stimuli.

By performing ERG recording, we identified 13 mutants with impaired photoresponse without morphology changes in compound eyes. Among them the two new mutants, *dmn* ^*K16109*^ and *rab6* ^*EP2397*^, reduced on- and off-transients. *Drosophila dmn* encodes p50/dynamitin, which is a subunit of dynactin complex and required for dynein-mediated transport [27]. The ERG of *dmn* ^*K16109*^ showed a normal light response except that on- and off-transient spikes were largely reduced, indicating impaired photoreceptor synaptic transmission (Fig 5A and 5B). Both excision of the P element and expression *dmn* in *dmn* ^*K16109*^ eye displayed a restored the on- and off- transient (Fig 5C and 5D). Similar as *dmn* ^*K16109*^, the ERG of *rab6* ^*EP2397*^ diminished on- and off- transient spike, while precise excision of P-element rescued this phenotype (Fig 5E and 5F). These results indicate Dmn and Rab6 are required for photoreceptor synaptic transmission.

### The *roh* mutant is defective in the production of rhodopsin

**Fig 6:**
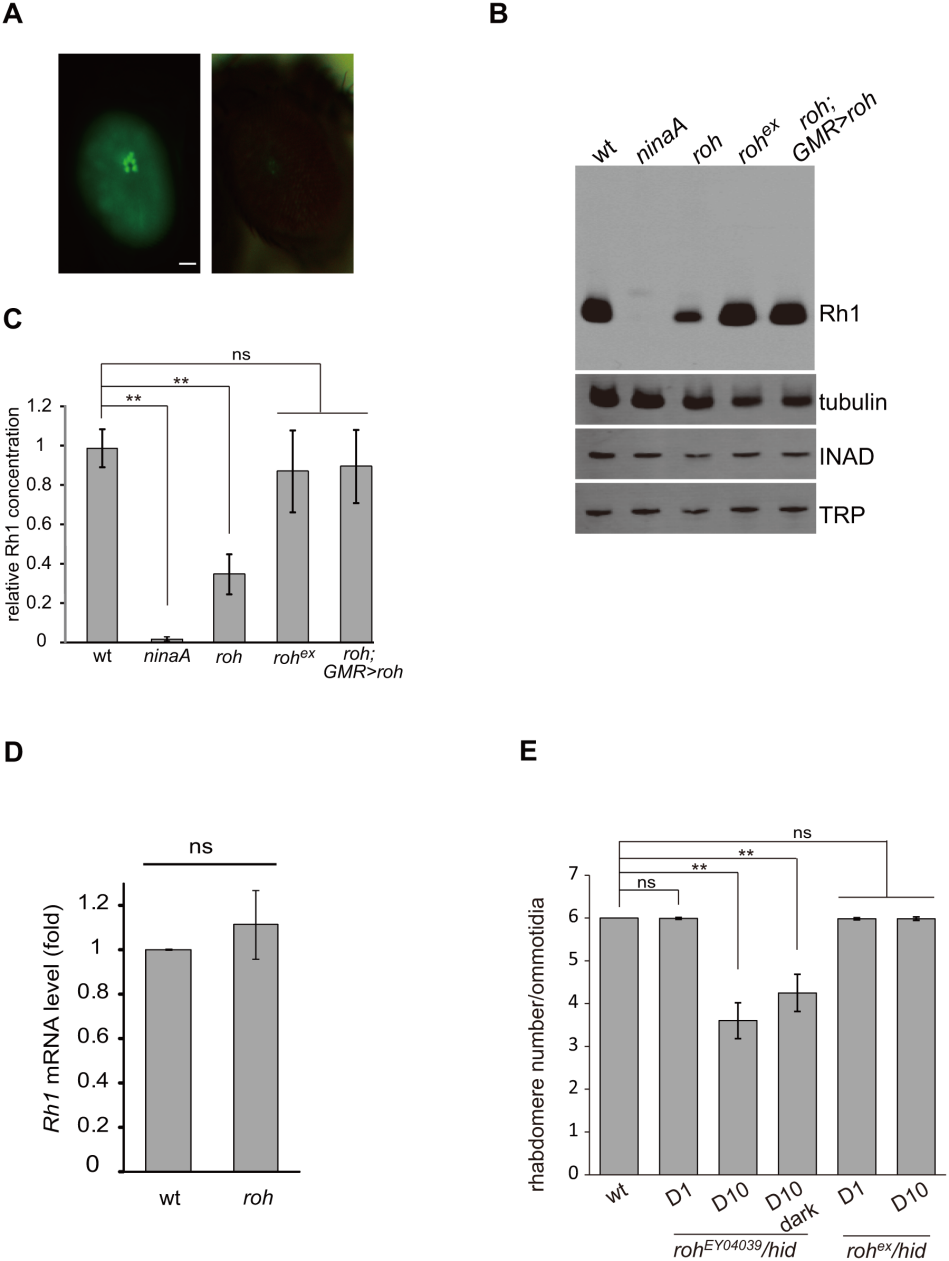
*roh* mutation results in Rh1 decrease. (A) GFP fluorescence of one-day old wild-type (left panel) and *roh* ^*EY04039*^ (right panel) flies showing a reduction in Rh1-GFP. Scale bar, 50 μm. (B) Western blotting showing decreased Rh1 levels in the *roh* ^*EY04039*^ mutant (*ey-flp Rh1*::*GFP; FRT42D roh* ^*EY04039*^ */ FRT42D GMR-hid CL*); the INAD and TRP levels are not affected compared to the wild-type. The reduction of Rh1 levels could be rescued in *roh* ^*ex*^ (*ey-flp Rh1*::*GFP; FRT42D roh* ^*ex*^ */FRT42D GMR-hid CL*) and *roh* ^*EY04039*^;*GMR>roh* (*ey-flp Rh1*::*GFP; FRT42D roh* ^*EY04039*^ */ FRT42D GMR-hid CL;GMR-gal4/UAS-roh*) flies. Flies less than 1 day old were used. (C) Quantification of relative Rh1 level in various genotypes: wt, *ninaA* ^*1*^, *roh* ^*EY04039*^, *roh* ^*ex*^ and *roh* ^*EY04039*^;*GMR>roh*. The Rh1 levels were normalized to tubulin. (D) Quantitative real-time PCR of wild-type (*ey-flp Rh1*::*GFP; FRT42D/ FRT42D GMR-hid CL*) and *roh* ^*EY04039*^ fly head with *rh1*-specific primers. The results are normalized to the expression level of *gpdh*. The graphs represent the means ± SD of three independent experiments. Error bars represent the SD. (E) Average rhabdomere numbers per ommatidia of the *roh* ^*EY04039*^ mutant under the indicated conditions. Each data point was based on examination of >60 ommatidia from >3 flies. Error bars represent the SDs. Asterisks indicate statistically-significant differences (one-way ANOVA and post-hoc Dunnett’s test; ns: not significant, **p < 0.01).

The maturation of rhodopsin is strictly regulated, and many mutations disrupting this process are known to cause reduced Rh1 accumulation [28, 29]. As we used Rh1-GFP to indicate Rh1 levels, we were able to target mutants that disrupt Rh1 homeostasis. The *CG30415* ^*EY04039*^ mutant eyes reduced Rh1-GFP fluorescence with normal morphology at one-day old (Fig 6A). We therefore named the *CG30415* gene as *roh* (*r* *eduction* *o* *f r* *h* *1*). We further checked if the mutation of roh specifically decreased Rh1 levels by performing western blots, and found that *roh* ^*EY04039*^ reduced the amount of Rh1 by 70% but expressed normal levels of INAD and TRP, two rhabdomere specific proteins (Fig 6B and 6C). Using real-time PCR, we found that the *rh1* mRNA levels were not changed in *roh* ^*EY04039*^ mutant eyes, which suggested that this reduction of Rh1 resulting from mutation of *roh* was not due to decreased *rh1* transcription (Fig 6D). Further, removal of the P-element and introduction of ROH back to photoreceptor cells by *GMR-Gal4/UAS-roh* restored the Rh1 levels of *roh* ^*EY04039*^ mutant eyes (Fig 6C). We next checked whether the *roh* ^*EY04039*^ mutant had altered localization of Rh1. Despite the reduced Rh1 signals, Rh1 exclusively localized to the rhabdomere in *roh* ^*EY04039*^ mutant eyes, independent of the light condition, suggesting that ROH is not required for Rh1 localization (S2 Fig). We next examined if loss of ROH leads to eventual retinal cell death using cornea optical neutralization assays. The *roh* ^*EY04039*^ mutant eye lost fluorescent rhabdomeres in an age-dependent manner, and remobilization of the P-element for the *roh* ^*EY04039*^ mutant (*roh* ^*ex*^) rescued this age-dependent retinal degeneration phenotype (Fig 6E). Moreover, the loss of rhabdomere fluorescence in the *roh* ^*EY04039*^ mutant eye was light-independent, as the *roh* ^*EY04039*^ mutant eye lost rhabdomeres in dark conditions as well (Fig 6E). These results suggest that ROH leads to retinal degeneration through downregulation of rhodopsin.

## Discussion

The *Drosophila* vision system has been served as important genetic system for searching the basis of and therapeutic treatments for human eye diseases. Two methods have been developed to make the entire eye homozygous for a mutation in otherwise heterozygous animals, satisfying the requirements for large scale genetic screening for essential genes [5, 6]. In one technique, with *minute* mutations integrated onto the marked FRT chromosome arms, which prevent the proliferation or survival of homozygous and heterozygous cells, the recombinant eyes are composed of more than 90% mutated chromosomes [6]. However, this method might not work well for some mutations that suppress growth rates. The *EGUF/hid* method generated flies with eyes composed exclusively of mutant clones by eliminating all photoreceptor cells not homozygous for the mutant chromosome arm. Here, we modified the *EGUF/hid* method by replacing *EGUF* with *ey-flp*, which abolished the fluorescence interruption by the pigmentation and simplified the genetic manipulation procedure [5]. It worth to mentioned that as the *ey-flp/hid* technique generates homozygous mutations in non-photoreceptor retinal cells as well, non-autonomous effects of these mutations on photoreceptor cell might be considered.

The time-consuming of ERG is a major concern for high-throughput F1 screen. Although rhabdomere and photoreceptor cell morphology based on the detection of simple deep pseudopupil (DPP) can be detected in living flies [30], the low sensitivity and the relatively transient lasting time prevent it widely to be used as a readout for high-throughput screen. Rh1-GFP introduced into flies, specifically marked the integrity and rhabdomeral morphology of photoreceptor cells and/or the amount of endogenous rhodopsin status. Through combining *Rh1*::*GFP* and *ey-flp/hid* technique, we can efficiently perform large scale screens for mutants of retinal degeneration and rhodopsin homeostasis.

The UCLA URCFG collection is a collection of P-element recessive lethal lines carrying FRT sequences, which has been successfully used by two retinal mosaic screenings for mutants of photoreceptor cell survival, eye development and rhodopsin [18–21]. One of the screenings used a two-color fluorescent imaging system to visualize the mosaic adult photoreceptor neurons with rhabdomere targeted Tomato, and identified multiple lines with defects in photoreceptor cell integrity including 4 mutations with apoptosis [18–21]. A similar retinal mosaic screening using Arrestin2::GFP to visualize endogenous Rh1 localization identified mutations of *PIG-U* (*Phosphatidylinositol glycan anchor biosynthesis*, *class U*) disrupting the transport of Rh1, along with several other mutations with rhabdomere morphological defects [18–21]. We screened UCLA URCFG collections and identified 14 retinal degeneration mutants, and 13 mutations with defective ERG. Among the 13 ERG mutants, 7 lines had defective phototransduction, and the other 6 lines only showed synaptic transmission defects. Importantly, 6 of 7 phototransduction mutants underwent retinal degeneration with the exception of *mmp2* mutant, which is correlated with the theory that most phototransduction mutations ultimately result in photoreceptor cell death. Therefore, the *Rh1*::*GFP ey-flp/hid*” method could be used to screen for mutants of phototransduction.

Beyond their primary function of supplying ATP, mitochondria play important roles in cell signaling events and apoptosis in eukaryotic metabolic processes. We found that mutations of two mitochondrial proteins, SCOX and Porin, cause light-dependent retinal degeneration. Although loss of either SCOX or Porin did not cause immediate cell death and abnormal mitochondria, both mutations are associated with deficiencies in mitochondrial respiration and ATP supply in mammalian and flies [31–33]. The ATP concentration in the rhabdomere is expected to be critical for normal phototransduction termination. Eye-enriched PKC encoded by the *inaC* (*inactivation nor afterpotential C*) locus is required for deactivation of the visual cascade by ATP-dependent phosphorylation of signaling molecules including TRP channels [34, 35]. Moreover, it has been reported that depletion of ATP rapidly activated the TRP and TRPL channels *in vivo*, suggesting that an ATP-dependent process is required to close the channels in phototransduction [36]. Mutations of *scox* and *porin* have similar defects in the termination of photoresponse, which might be caused by reduction of ATP due to impairment of mitochondria function, rather than directly functioning in phototransduction [26]. Moreover, mutants that failed to terminate photoresponse likely keep excessive Ca^2+^ influx, and accumulate stable Rh1/Arr2 complexes during light responses, which potentially leads to retinal degeneration [37]

Rab6 is present in the Golgi apparatus and is involved in protein transport via the Golgi apparatus [38, 39]. A dominant negative Rab6 expression leading to a reduction of mature Rh1 levels suggested a role of Rab6 in rhodopsin transport [40]. However, only the synaptic function of Rab6 was reported in a recent loss function study of Rab6 [41]. Consistent with these results, we found that mutation of rab6 does not reduce Rh1 levels or alter its distribution, which forces questioning of the supposed role of Rab6 in rhodopsin maturation. Transport of neuronal vesicles along the microtubules is known to require Dynein as a molecular motor, and the dynactin complex functions as dynein cargo adaptor that participates in this neuronal vesicles transport process [42]. The mutations in *p150* ^*Glued*^, a large subunit of dynactin, caused defects in vesicular transport and neurotransmitter release in motor neurons of both mice and flies [43, 44]. Here, we observed that a mutation in the *dmn* gene that encodes the dynactin subunit 2, dynamitin, disrupted synaptic transmission in photoreceptor cells, providing evidence that the dynactin complex has a critical function in neuronal cell synaptic transmission. Rab6 has been reported to interact with the dynamitin complex and thus function in an important role in microtubule-dependent retrograde trafficking from the endosome to the Golgi and from the Golgi to the ER [45–47]. Given that both *rab6* ^*EP2397*^ and *dmn* ^*K16109*^ display similar lack of on- and off- transients in the ERG recording profile, Rab6 might function with dynamitin in neuronal vesicle transport along the microtubules.

The first mutation identified to cause retinal degeneration was *Drosophila ninaE*, which encode the major rhodopsin, Rh1 [48–50]. Later evidence in human patients showed that mutations in rhodopsin and related genes are major causes of retinitis pigmentosa, the most common form of retinal degeneration disease [51, 52]. As our screening system takes advantage of Rh1-GFP as marker, the key factors affecting Rh1 synthesis and maturation can be targeted. We identified a new factor ROH related to Rh1 synthesis, and suggested that ROH is required for Rh1 biosynthesis and photoreceptor cell survival. However, as ROH does not contain any obvious protein motifs and exhibits no significant homology to any known protein in common databases, the mechanisms of how ROH affects Rh1 biosynthesis need to be further investigated.

The *Drosophila* vision system is a powerful model for the study of neural development, signal networks and photoresponse maintenance; studies based on this model system have provided important insights in the pathogenesis of several neurodegenerative diseases [53]. Several neurodegenerative diseases models, including Parkinson disease, Alzheimer's disease, Polyglutamine Diseases, and Amyotrophic Lateral Sclerosis disease were established in flies by inducing degenerative cell death of photoreceptor neurons in the compound eye [54–59]. Our method could represent a powerful tool for genetic screening of modifiers of neurodegenerative diseases.

## Experimental Procedures

### Fly Stocks

The following stocks were used: *ey-flp Rh1*::*GFP;GMR-hid CL FRT40A/Cyo hs-hid*, *ey-flp Rh1*::*GFP; FRT42D GMR-hid CL/Cyo hs-hid*, *ey-flp Rh1*::*GFP;GMR-hid CL FRT80B/TM3 hs-hid*, *ey-flp Rh1*::*GFP;GMR-hid CL FRT80B/TM3 hs-hid*, *ey-flp Rh1*::*GFP; FRT82B GMR-hid CL /TM3 hs-hid*, *ey-flp Rh1*::*GFP;ninaA* ^*1*^ *40A*, *ey-flp Rh1*::*GFP;trp* ^*343*^, *ey-flp Rh1*::*GFP;Hdc* ^*P217*^, and *hs-flp;ubi-RFP FRT40A*. The *P{ry[+t7*.*2] = ey-FLP*.*N}2* (*ey-flp*), *longGMR-gal4*, and *UAS-dmn* flies were obtained from the Bloomington stock center. The UCLA URCFG collections were obtained from the Kyoto Drosophila Genetic Resource Center. The *Scox* ^*ex*^, *porin* ^*ex*^, *dmn* ^*ex*^, *rab6* ^*ex*^, and *roh* ^*ex*^ flies were generated by precise excision of the P-element lines *scox* ^*EY05333*^, *porin* ^*k05123*^, *dmn* ^*K16109*^, *rab6* ^*EP2397*^ and *roh* ^*EY04039*^, respectively. Briefly, P-element lines were mobilized by genetically introducing transposase using the Δ2–3 line, and precise excision lines were confirmed by DNA sequencing.

### Generation of transgenic flies

To express GFP-labeled Rh1 under control of the *ninaE* (*rh1*) promoter, a GFP tag was added to the C terminus of the *rh1* cDNA sequence and subsequently subcloned into the *pcNX* vector between the NotI and XbaI sites [60]. The *w* gene on the construct was subsequently knocked out by introducing a frame-shift mutation in its coding region. The construct was injected into *w* ^*1118*^ embryos, and transformants were identified on the basis of GFP fluorescence. The *CG30415* cDNA molecule was amplified from the cDNA clone GH51119, and was subcloned into the *puast-attB* vector. The construct was injected into *M{vas-int*.*Dm}ZH-2A;M{3xP3-RFP*.*attP}ZH-86Fb* embryos, and transformants were identified on the basis of eye color.

#### Generation of the CoIV and Tom20 Antibodies

Polyclonal antibodies against *Drosophila* Cytochrome c Oxidase Subunit IV (CoIV) were generated by immunizing a rabbit with a synthetic peptide (IIDLEINPVTGLTSKWDYENKKW) conjugated to KLH. Polyclonal antibodies against Drosophila Tom20 were generated by immunizing a rat with a synthetic peptide (QEFGNRAAEGNDGPIVLGQS) conjugated to KLH. The specificity of the antibodies was testified by staining adult thoraxes (S1 Fig).

### Fly imaging and optical neutralization assay

Flies were anaesthetized on a CO_2_ pad, and fluorescence and bright-field images were taken with Leica M165 FC Fluorescent Stereo Microscope. To perform optical neutralization assays, the heads of flies were cut off, and immerged into mineral oil in an orientation with eyes on the upper side. The samples were examined with a Nikon Ni-U fluorescent microscope.

### Immunohistochemistry

Imaginal discs or adult thorax muscles were dissected in PBS solution (PH 6.8) and fixed in 4% paraformaldehyde in PBS buffer for 30 min. Eye discs or muscles were incubated in diluted primary antiserum, rabbit monoclonal anti-CoIV (1:200) and rat anti-Tom20 (1:200). Anti-rabbit and rat labeled secondary antibodies with Alexa 488, 568, or 647 (1:500) (Invitrogen) were used as secondary antibodies, and Phalloidin 650 (Thermo Scientific) was added to label actin filaments.

For eye section staining, semi-sectioned fly heads were fixed in 4% paraformaldehyde in PBS buffer on ice for 2 hours, and then embedded in LR White resin. Thin sections were prepared at a depth of ~30 μm and incubated with mouse monoclonal anti-Rh1 (1:200) and Rat anti-TRP (1:200) as primary antibodies and Alexa 647 or 750 labeled secondary antibodies (Invitrogen). Samples were examined and images were recorded using a Nikon confocal microscope. The acquired images were processed with Photoshop software.

### Western blots

To perform western blots, fly heads were homogenized in SDS sample buffer with a Pellet Pestle (Kimble/Kontes). The proteins were fractionated by SDS-PAGE and transferred to Immobilon-P transfer membranes (Millipore) in Tris-glycine buffer. The blots were probed with mouse Tubulin primary antibodies (1:2000 dilution, Developmental Studies Hybridoma Bank), mouse Rh1 antibodies (1:2000 dilution, Developmental Studies Hybridoma Bank) and Rat anti-INAD (1:2000, C. Montell lab), followed by IRDye 680 goat anti-mouse IgG (LI-COR) and IRDye 800 goat anti-Rat IgG (LI-COR) as the secondary antibodies. The signals were detected with the Odyssey infrared imaging system (LI-COR).

### Electroretinogram Recordings

ERG recordings were performed as described previously [61]. A Newport light projector (model 765) was used for stimulation. ERG signals were amplified with a Warner electrometer IE-210 and recorded with a MacLab/4 s A/D converter and the clampelx 10.2 program (Warner Instruments).

### Transmission electron microscopy

Adult fly heads were dissected and fixed in paraformaldehyde/glutaraldehyde and osmium tetroxide solutions, dehydrated with an ethanol series, and embedded in LR White resin as described previously [62]. Thin sections (80 nm) were prepared at a depth of 30–40 μm and were examined using a transmission electron microscope (model 1230; JEOL). The images were acquired using a bottom-mount charge-coupled device camera (Ultrascan 1000; Gatan, Inc.). Digital Micrograph software (Gatan, Inc.) was used to convert images into TIFF files.

## Supporting Information

S1 Fig Reduced CoIV levels in *scox* mutant cells. (A) Verification of anti-CoIV and anti-Tom20 antibodies. Muscle fibers and mitochondria were organized into parallel stripes within indirect flying muscles in the thorax. Mitochondria of adult thoraxes were stained with anti-CoIV and anti-Tom20 antibodies, while muscle fibers were visualized by Phalloidin staining. Scale bar, 10 μm. (B) Imaginal disc staining of *hs-flp; scox* ^*EY05333*^ *FRT40A/ubi-RFP FRT40A* by anti-CoIV (upper panel) and anti-Tom20 (lower panel) antibodies. RFP negative cells that are circled are *scox* homozygous mutant cells. Scale bar, 10 μm.
(EPS)

S2 Fig The *roh* mutation did not affect the rhabdomeral localization of Rh1. (A) adult eye sections were stained by anti-Rh1 (red) and anti-TRP (green) antibodies. One day-old *roh* ^*EY04039*^ */hid* (*ey-flp Rh1*::*GFP; roh* ^*EY04039*^ *FRT42D/GMR-hid CL FRT42D*) and *roh* ^*ex*^ */hid* (*ey-flp Rh1*::*GFP; roh* ^*ex*^ *FRT42D/GMR-hid CL FRT42D*) flies were raised either under a 12 hr light/12 hr dark cycle or in absolute dark conditions. Scale bar, 50 μm.
(EPS)

S1 Table Phenotypes of 667 lethal lines. (XLS)
